# Efficacy of dulaglutide on vascular health indexes in subjects with type 2 diabetes: a randomized trial

**DOI:** 10.1186/s12933-020-01183-5

**Published:** 2021-01-04

**Authors:** Antonino Tuttolomondo, Anna Cirrincione, Alessandra Casuccio, Alessandro Del Cuore, Mario Daidone, Tiziana Di Chiara, Domenico Di Raimondo, Vittoriano Della Corte, Carlo Maida, Irene Simonetta, Stefania Scaglione, Antonio Pinto

**Affiliations:** 1 Department of Promoting Health, Maternal-Infant, Excellence and Internal and Specialized Medicine (ProMISE) G. D’Alessandro, University of Palermo (Italy), Piazza delle Cliniche n.2, 90127 Palermo, Italy; 2 Internal Medicine and Stroke Care Ward, Policlinico ‘P. Giaccone’, Palermo, Italy; 3 PhD Programme in Molecular and Clinical Medicine, University of Palermo, Piazza delle Cliniche n.2, 90127 Palermo, Italy

**Keywords:** Dulaglutide, Vascular health, Diabetes

## Abstract

**Background:**

Recent cardiovascular outcome trials have shown significant reductions in major cardiovascular (CV) events with glucagon-like peptide (GLP)-1 receptor agonists. Additionally, adjunctive surrogates for cardiovascular risk validated by some studies include arterial stiffness and endothelial function indexes. To date, no randomized trial has addressed the possible effects of antidiabetic interventional drugs such as GLP1 agonists on endothelial and arterial stiffness indexes as surrogate markers of vascular damage.

**Aims:**

We aimed to evaluate metabolic efficacy and surrogate vascular efficacy endpoints of once-weekly dulaglutide (1.5 mg) plus traditional antidiabetic treatment compared with traditional antidiabetic treatment alone in subjects with type 2 diabetes.

**Methods:**

Men and women (aged ≥ 50 years) with established or newly detected type 2 diabetes whose HbA1c level was 9.5% or less on stable doses of up to two oral glucose­ lowering drugs with or without basal insulin therapy were eligible for randomization. Subcutaneous dulaglutide was initiated at the full dose (1.5 mg/day weekly). Arterial stiffness (PWV: pulse wave velocity and augmentation index) and endothelial function (RHI: reactive hyperaemia index) were evaluated at baseline and at three-month and nine-month examination visits. At each visit (at 3 and 9 months), the subjects were also evaluated for glycaemic variables such as fasting plasma glucose (FPG) and HbA1c and lipid variables such as total cholesterol, LDL cholesterol, HDL cholesterol and triglyceride levels.

**Results:**

At the three-month follow-up, the subjects treated with dulaglutide showed significantly lower serum levels of FPG and HbA1c than control subjects treated with conventional therapy. At the 9-month follow-up, subjects treated with dulaglutide showed significant lower values of the mean diastolic blood pressure, BMI, total serum cholesterol, LDL cholesterol, FPG, HbA1c and PWV and higher mean RHI values than control subjects treated with conventional therapy.

**Conclusions:**

Our randomized trial showed that subjects with type 2 diabetes treated with conventional therapy plus 1.5 mg/day of subcutaneous dulaglutide compared with subjects treated with conventional therapy alone showed favourable metabolic effects associated with positive effects on vascular health markers such as arterial stiffness and endothelial function markers. These findings are consistent with previous study findings indicating the strict relationship between cardiovascular risk factors such as systolic blood pressure, total serum cholesterol and LDL levels and cardiovascular events and vascular health surrogate markers.

## Background

Glucagon-like peptide-1 (GLP-1) is a member of an endogenous class of incretin hormones synthesized in intestinal epithelial cells in response to gastrointestinal nutrients [1]. GLP-1 enhances glucose-dependent secretion of insulin [2, 3], inhibits glucagon secretion [4], slows gastric emptying [5] and reduces food intake [6, 7].

Dulaglutide (Eli Lilly and Co., Indianapolis, IN, USA), a long-acting GLP-1 receptor agonist [8], mimics some endogenous GLP-1 effects. Dulaglutide has been approved in the USA and European Union at once-weekly doses of 0.75 and 1.5 mg as a subcutaneous injection to improve glycaemic control in patients with type 2 diabetes.

In global clinical trials completed to date, dulaglutide (1.5 mg) is superior to metformin, sitagliptin and exenatide twice daily and non-inferior to liraglutide (1.8 mg) for glycated haemoglobin (HbA1c) changes [9–12].

Endothelial dysfunction and an abnormal vascular structure may represent complications of diabetes. Substantial clinical and experimental evidence suggests that both diabetes and insulin resistance cause a combination of endothelial dysfunction, which may diminish the anti-atherogenic role of the vascular endothelium [13–15].

The initiation and progression of atherosclerosis may originate from impaired endothelial function detected at the earliest stages of the syndrome. The elements of the metabolic syndrome and accelerated phases of atherogenesis are often silent partners that present many years before the onset of type 2 diabetes mellitus. The ability to detect subclinical vascular disease, reflected as multiple factors that impair arterial wall integrity, offers new tools to refine cardiovascular risk stratification [16]. Endothelial dysfunction in peripheral arteries is evaluated by forearm flow-mediated vasodilation (FMD) [17]. However, this technique offers findings that can widely vary due to technical problems, limiting its use as a fully standardized method [18].

Kuvin et al. [19] described a new method to evaluate endothelial dysfunction, called reactive hyperaemia peripheral arterial tonometry (RH-PAT). This method measures the hyperaemic response non-invasively. The Framingham Heart Study reported that reactive hyperaemia index (RHI) is inversely related to various cardiovascular risk factors [20], confirming the usefulness of this test.

Arterial stiffness indexes, such as the augmentation index (AIx) and pulse wave velocity (PWV), have been reported as surrogate cardiovascular markers [21], whereas endothelial dysfunction has been reported as an associated factor [20].

Adjunctive surrogates for cardiovascular risk validated by some studies more recently [22, 23] are arterial stiffness and endothelial function indexes.

Recent cardiovascular outcome trials have shown significant reductions in major cardiovascular (CV) events using these glucagon-like peptide (GLP)-1 receptor agonists [14–16, 24].

Progressive separation of the treatment and placebo curves, starting clearly between 12 and 18 months of the trial period, as well as significant reductions in the risk of myocardial infarction and stroke, indicate that the beneficial CV effects observed using GLP-1 receptor agonists could be due to an anti-atherogenic effect [25].

The use of glucose-lowering agents is an effective strategy to prevent vascular complications, but the extent to which it can reduce CV complications is less certain. Glucagon-like peptide-1 (GLP-1) agonists are potent glucose-lowering agents but also have potentially beneficial effects on other traditional [body weight, blood pressure (BP), and LDL cholesterol] and non-traditional risk factors (low-grade inflammation and endothelial dysfunction).

Our group recently reported that, in some cohorts of patients, such as patients with diabetic foot [26], subjects with NAFLD [27] and patients with acute ischaemic stroke [28], higher mean values of arterial stiffness markers (PWV, AIx) and lower mean values of reactive hyperaemia indexes (RHI) may be considered surrogate markers of vascular damage.

A previous recent study on a small sample of patients showed that once-weekly dulaglutide was comparable to once-daily liraglutide in terms of its effects on oxidative stress and endothelial function, as measured by RHI [29].

To date, no randomized trial has addressed the possible effects of antidiabetic interventional drugs such as GLP1 agonists on endothelial function, as measured by RHI, and arterial stiffness indexes, such as surrogate markers of vascular damage.

## Study hypothesis

Our study hypothesis is that the anti-atherogenic effect and consequent beneficial cardioprotective profile of dulaglutide may be related to its metabolic efficacy and possible efficacy on vascular health indexes such as endothelial dysfunction and arterial stiffness markers.

## Objective of the study

In the present randomized trial, we compared once-weekly dulaglutide (1.5 mg) plus traditional antidiabetic treatment with traditional treatment alone using surrogate vascular efficacy endpoints such as endothelial function and arterial stiffness indexes and metabolic efficacy endpoints.

## Aims and endpoints

The first aim of the study was to show the superiority of dulaglutide vs. traditional treatment for vascular health indexes such as markers of arterial stiffness (PWV, AIx) and endothelial function such reactive hyperaemia index (RHI) changes from baseline at the three- and nine-month follow-ups.

The second aim of the study was to show the superiority of dulaglutide vs. traditional treatment for changes in metabolic variables (*fasting plasma glucose*, HbA1c, *total cholesterol, LDL-cholesterol, HDL-cholesterol, and triglyceride levels*) from baseline at the three- and nine-month follow-ups.

The vascular efficacy endpoints were as follows:

- change from baseline in the RHI value at the 3- and 9-month follow-ups
- change from baseline in PWV at the 3- and 9-month follow-ups
- change from baseline in AIx at the 3- and 9-month follow-ups

The metabolic efficacy endpoints were as follows:

- change from baseline in body weight at the 3- and 9-month follow-ups
- change from baseline in BMI at the 3- and 9-month follow-ups
- change from baseline in FPG at the 3- and 9-month follow-ups
- change from baseline in HbA1c at the 3- and 9-month follow-ups
- change from baseline in the serum total cholesterol at the 3- and 9-month follow-ups
- change from baseline in the serum LDL cholesterol at the 3- and 9-month follow-ups
- change from baseline in the serum HDL cholesterol at the 3- and 9month follow-ups

## Materials and methods

### Study design and participants

This study was a single-centre, 9-month randomized trial designed to evaluate the efficacy of antidiabetic traditional treatment + dulaglutide compared with traditional antidiabetic treatment alone in patients with type 2 diabetes.

Traditional antidiabetic treatment was considered treatment with metformin or metformin plus sulfonylurea or basal insulin plus metformin.

Men and women (aged ≥ 50 years) with established or newly detected type 2 diabetes whose HbA1c level was 9.5% or less on stable doses of up to two oral glucose­lowering drugs with or without basal insulin therapy were eligible.

The key exclusion criteria for the screened patients were as follows: type 1 diabetes, previous GLP-1 receptor agonist treatment, treatment with more than half of the sulphonylurea maximum dose at screening, chronic systemic glucocorticoid use, or gastric emptying abnormality.

Further exclusion criteria were an eGFR value less than 15 mL/min per 1.73 m², cancer in the last 5 years, severe hypoglycaemia in the last year, a life expectancy of less than 1 year, a coronary or cerebrovascular event within the last 2 months, and plans for revascularization.

A common protocol was approved by the institutional review board, and the study was performed in accordance with the principles of the Declaration of Helsinki and Good Clinical Practice [30].

Each patient provided written informed consent before participation.

The trial has been registered on Clinical Trials.gov [ClinicalTrials.gov Identifier: NCT03824002].

We used the revised criteria of the American Diabetes Association to diagnose type 2 diabetes (T2DM)—a fasting blood glucose value ≥ 126 mg/dl or a clinically based algorithm that considered presenting weight and symptoms, age at onset, onset of insulin treatment, family history and history of ketoacidosis [31].

Hypertension was diagnosed according to the 2013 ESC-ESH criteria [32].

Dyslipidaemia was defined as a TG level ≥ 150 mg/dL and HDL cholesterol level < 40 mg/dL.

regardless of the patient’s gender [33]

### Randomization

After a 2-week placebo screening and run-in period, eligible patients were randomly assigned to undergo traditional antidiabetic treatment plus dulaglutide or traditional antidiabetic treatment alone using a computer-generated random sequence (1:1) with dulaglutide administered once weekly for 9 months. The investigators involved in clinical data collection and the measurement of outcome variables were not directly involved in the patients’ treatment and were masked to the randomization process. The randomization code was maintained only at the central data facility and was not broken until all data analysis was complete.

### Procedures

Subcutaneous dulaglutide was initiated at the full dose (1.5 mg/day weekly).

During the treatment period, participants in the dulaglutide group were instructed to inject the study drug on the same day at approximately the same time each week. The participants were visited at 2 weeks, 3 months, and 9 months. At every visit, assessments included cardiovascular events, adverse events, vital signs, and periodic questionnaires, laboratory test electrocardiograms (ECGs) and vascular damage index assessment (see above). Investigators were advised to promote a healthy lifestyle and manage glucose concentrations according to local guidelines. The management of blood pressure, lipids, other cardiovascular risk factors, and medical conditions was at the discretion of the study investigator.

Hypoglycaemia was defined as a blood glucose concentration ≤ 3.9 mmol/L. Severe hypoglycaemia was defined as an episode that required the assistance of another person to actively administer carbohydrate, glucagon or other resuscitative actions. The patients were allowed to initiate rescue therapy for severe, persistent hyperglycaemia according to predefined thresholds of fasting blood glucose for at least 2 weeks with no readily identifiable cause.

## Methods of measurement

### Measurement of the heart rate at rest

According to the Consensus Panel of the European Society of Hypertension [33] the following information should be provided in studies reporting heart rate data: (i) resting period before measurement; (ii) environmental conditions; (iii) method of measurement; (iv) number of measurements; (v) duration of measurement; (vi) body position; and (vii) nature of the observer. The table shows the recommendations provided by the panel regarding how the heart rate should be measured.

To achieve a stable haemodynamic condition, the patient should rest for at least 5 min, although subjects with a pronounced white-coat reaction may require a longer period. The duration of measurement ranges from 15 s to 1 min in different studies. According to the European consensus panel [33], 30 s is sufficient to obtain a reliable estimate of the heart rate because, in most patients, 30 to 40 cardiac cycles can be averaged. In subjects with a very low heart rate, a longer period may be necessary. Two measurements were considered sufficient for a reliable estimate of the resting heart rate in most patients.

### Measurement of the blood pressure

According to the Consensus Panel of the European Society of Hypertension [33], the recommended steps to measure blood pressure in our enrolled patients were:

- The patients were on a sitting position (comfortable, relaxed, legs uncrossed, feet resting on the floor) for 5 min before obtaining the measurement.
- The arm should be supported at the level of the heart and slightly flexed at the elbow.
- The BP cuff is placed with the bladder midline over the brachial artery pulsation.

The lower border of the cuff should be approximately 2.5 cm above the antecubital crease.

- To determine the inflation level, the radial artery is palpated and the cuff is rapidly inflated until the pulse disappears;, this pressure was recorded from the manometer and 30 mmHg was added to the value.
- The cuff is deflated, followed by a delay for 15–30 s.
- The stethoscope is placed lightly over the brachial artery.
  - The Korotkoff sounds are best heard with the bell of the stethoscope because they are relatively low in pitch.
  - A proper seal is confirmed.
- The cuff is inflated rapidly to the predetermined inflation level (see step 4).
- The bulb’s screw is turned counter-clockwise to deflate slowly at a rate of 2–3 mmHg/s.
- The level is recorded at which the sounds of at least two consecutive beats are heard (Korotkoff phase I). This value represents the patient’s systolic BP.
- The cuff is allowed to deflate until the sounds become muffled and disappear (Korotkoff phase V). This value represents the patient’s diastolic blood pressure.
- The cuff is deflated rapidly.
- The systolic and diastolic levels are read to the nearest 2 mmHg.
- The blood pressure, arm used, arm position, and cuff size are recorded.
- The operator waits ≥ 2 min if measurements are repeated.

### PWV measurement

Carotid-femoral PWV was assessed in the supine position using an automatic device (SphygmoCor version 7.1) that evaluated the time delay between the rapid upstroke of the carotid and femoral artery pulse waves. The distance between the two arterial points was measured using a tape measure on the surface of the body. PWV was valuated as the distance travelled by the arterial pulse wave (m) divided by the time delay between the two arterial points (s), expressed as metres per second.

### Pulse wave analysis

We used applanation tonometry to record the radial artery pressure waveform continuously, and mean values ≥ 2 screens of pulse waves of good quality were used for analysis. Considering the collected data, an averaged radial pressure waveform was created and a corresponding aortic pressure waveform and BP were calculated using the validated transfer function (SphygmoCor version 7.1). The aortic pressure waveform was utilized to calculate the AIx (difference in height between the first and second systolic peaks, expressed as a percentage of PP).

### RH-PAT

The principle of RH-PAT has been described previously [18]. Briefly, a blood pressure cuff was positioned on 1 upper arm, while the contralateral arm served as a control. PAT probes were positioned on one finger of each hand. After a 5-min equilibration period, the cuff was inflated to 60 mm Hg above the systolic pressure or 200 mm Hg for 5 min and then deflated to induce reactive hyperaemia. Reactive hyperaemia is a temporary increase in blood flow in an area due to induced ischaemia and reflects ‘the health state’ of the endothelium.

The RH-PAT data were digitally analysed online using Endo-PAT2000 software version 3.0.4. The RH-PAT index (RHI: reactive hyperaemia index) reproduces the range of reactive hyperaemia and is measured as the ratio of the mean amplitude of the PAT signal over 1 min starting 1.5 min after cuff deflation (control arm, A; occluded arm, C) divided by the mean amplitude of the PAT signal of a 2.5-min time period before cuff inflation (baseline) (control arm, B; occluded arm, D). Thus, RH-PAT index (RHI) = (C/D)/(A/B) × baseline correction. A value of RHI < 1.67 indicated endothelial dysfunction.

### Vascular damage marker evaluation

PWV, AIx and RHI were measured at admission and every three- and nine-month follow-up visit assessment.

### Statistical analysis

The sample-size (performed by IBM SPSS SamplePower 3) of at least 40 randomized patients was selected to provide > 99% power to demonstrate superiority of dulaglutide respect to conventional therapy alone. This assumed a true mean difference in metabolic and vascular variable mean value change from baseline between dulaglutide and placebo of 0.8%, a common standard deviation of 1.1%, a one-sided significance level of 0.025, and a 9% drop-out rate between randomization and week [27]. Moreover, the given sample-size provided at least 90% power to confirm non-inferiority of dulaglutide to liraglutide with a margin of 0.4%.”

Continuous data were expressed as the means ± SD, unless otherwise specified. Intergroup differences were assessed by chi-squared test or Fisher’s exact test, as needed for categorical variables, and by independent Student’s t test for continuous parameters if the data were normally distributed. Intragroup differences were performed by repeated measures analysis of variance (ANOVA), and post-hoc analysis with Tukey’s test was used to determine if there were any intra-group differences in pairs.

The data were analysed using IBM SPSS Software 22 version (IBM Corp., Armonk, NY, USA). All p-values were two-sided, and p < 0.05 was considered statistically significant.

## Results

From April 2, 2017 to April 12, 2019, 124 patients entered the study: 60 were randomized to treatment with the study drug (56 completed 3 months and 9 months of treatment), and 64 were control subjects. Twelve patients (4 in the group with traditional treatment plus dulaglutide and 8 in the control group) discontinued the study, with ‘withdrawal by subject’ being the most common reason.

The patient demographics and baseline characteristics were similar between the groups (Table 1).

**Table 1:** general, demographic and laboratory variables in subjects treated with dulaglutide vs. controls ( conventional therapy)

Variables	Subjects treated with conventional therapy + dulaglutide (n = 56)		Controls (conventional therapy) (n = 56)	p
Age (years) (mean ± SD)	69.7 ± 8.6		67.6 ± 5.1	0.111
M/F (n/%)	24/32 (42.8/57.2)		21/35 (37.5/62.5)	0.700
SBP (mm/Hg) (mean ± SD)	137.8 ± 12.1		133.9 ± 12.3	0.087
DBP (mm/Hg) (mean ± SD)	76.3 ± 12.3		79.8 ± 7.7	0.071
Weight (Kg) (mean ± SD)	75.7 ± 8.5		76.6 ± 7.5	0.562
BMI (kg/m²) (mean ± SD)	27.6 ± 3.4		27.9 ± 3.2	0.563
HR (bpm) (mean ± SD)	76.6 ± 11.1		79.3 ± 15.8	0.299
Total cholesterol (mg/dL) (mmol/L) (mean ± SD)	160.9 ± 20.5/4.2 ± 0.5		167.8 ± 25.8/4.3 ± 0.7	0.121
HDL cholesterol (mmol/L) (mean ± SD)	37.4 ± 4.3/0.9 ± 0.1		37.5 ± 4.5/0.9 ± 0.2	0.961
LDL cholesterol (mmol/L) (mean ± SD)	102.7 ± 11.6/2.6 ± 0.3		104.5 ± 13.5 /2.7 ± 0.4	0.439
Triglycerides (mmol/L) (mean ± SD)	139.2 ± 13.6/1.6 ± 0.2		138.9 ± 13.6/1.6 ± 0.2	0.910
FPG ( mg/dL/ mmol/L (mean ± SD)	147.6 ± 32.6 /8.2 ± 1.2		144.7 ± 31.5 /8.1 ± 1.8	0.626
HBa1C ( %) (mean ± SD)	7.4 ± 0.7		7.2 ± 0.6	0.101
eGFR CKD-EPI (mL/min per 1·73 m²) (mean ± SD)	77.4 ± 11.2		76.1 ± 16.9	0.640
Microalbuminuria (mg/24 h) (mean ± SD)	80.9 ± 20.9		73.1 ± 29.2	0.107
RHI (mean ± SD)	1.7 ± 0.4		1.8 ± 0.7	0.275
PWV (m/s) (mean ± SD)	11.2 ± 0.9		10.9 ± 0.8	0.193
Aix ( %) (mean ± SD)	105.6 ± 6.9		103.9 ± 4.4	0.121
Hypertension (n/%)	45/80.3		35/62.5	0.700
Diabetes (n/%)	56/100		56/100	–
Duration of diabetes (years) (mean ± SD)	10.4 ± 3.3		10.2 ± 4.0	0.773
Dyslipidaemia (n/%)	32 (57.1)		29 (51.8)	0.704
Hypercholesterolaemia (n/%)	31 (55.3)		27 (48.2)	0.570
Smoking (n/%)	12 (21.4)		13 (23.2)	1.0
Microalbuminuria	12 (21.4)		11 (19.6)	1.0
Macroalbuminuria	4 (7.1)		3 (5.4)	1.0
Metformin (n/%)	50 (89.3)		49 ( 87.5)	1.0
Sulfoniluree (n/%)	31 (55.3)		30 (53.6)	1.0
Insulin (n/%)	12 (21.4)		11 (19.6)	1.0
ACEi (n/%)	28 (50.0)		32 (57.1)	0.569
Beta blockers (n/%)	28 (50.0)		24 (42.8)	0.570
CCB (n/%)	21 (37.5)		22 (39.3)	1.0
Antiplatelets (n/%)	23 (41.1)		18 (32.1)	0.432
Anticoagulants (n/%)	30 (53.6)		30 (53.6)	1.0
Diuretics(n/%)	27 (48.2)		30 (53.6)	0.705
Statins (n/%)	22 (39.3)		16 (28.6)	0.318

Data are expressed as mean (SD), n (%)

*SBP* systolic blood pressure, *DBP* diastolic blood pressure, *BMI* body mass index, *HR* heart rate, *LDL* low density lipoprotein, *HDL* high density lipoprotein, *FPG* fasting plasma glucose, *HBa1C* glycated hemoglobin, *PWV *pulse wave velocity, *AIX* augmentation index, *E-GFR* estimated glomerular filtration rate, *SGLT2 *sodium-glucose *co*-transporter-2, *ACE* angiotensin-converting enzyme, *ARB *angiotensin-receptor blocker, *HbA1c *glycated haemoglobin A1c, *eGFR* estimated glomerular filtration rate

At the end of the randomization phase, 56 patients completed the study for each group.

At baseline, the patients treated with dulaglutide plus traditional therapy and control subjects treated with traditional therapy showed no significant difference regarding age (69.7 ± 8.6 years vs. 67. 6 ± 5.1 years, respectively; p = 0.111), duration of diabetes (10.4 ± 3.3 years vs. 10.2 ± 4.0 years, respectively; p = 0.773), mean fasting plasma glucose (FPG) (147.6 ± 32.5 mg/dl vs. 144.6 ± 31.5 mg/dL, respectively; p = 0.626), mean HbA1c (7.4 ± 0.7% ± 7.2 ± 0.6%, respectively; p = 0.101), mean total cholesterol (160.9 ± 20.5 mg/dL vs. 167.8 ± 25 mg/dL mmol/L; p = 0.121), mean HDL cholesterol (37.4 ± 4.3 mg/dL vs. 37.5 ± 4.5 mg/dL, respectively; p = 0.961), mean LDL cholesterol (102.7 ± 11.6 mg/dL/2.65 ± 0.3 mmol/L vs. 104.5 ± 13.6 mg/dL/2.7 ± 0.35 mmol/L, respectively; p = 0.439), mean triglyceride level (139.2 ± 13.6 mg/dL vs. 138.90 ± 13.58 mg/dL, respectively; p = 0.910), mean RHI (1.7 ± 0.4 vs. 1.8 ± 0.7, respectively; p = 0.275), and PWV (11.2 ± 0.91 m/sec vs. 10.9 ± 0.8 m/s, respectively; p = 0.193) (see Table 1).

The subjects treated with dulaglutide and control subjects treated with traditional therapy showed no significant differences regarding the other clinical and laboratory variables at baseline.

### Efficacy

At the three-month follow-up, the subjects treated with dulaglutide, compared with control subjects treated with conventional therapy, showed no significant difference concerning SBP, DBP, BMI, total cholesterol, LDL-cholesterol, HDL-cholesterol, triglycerides, GFR CKD-EPI, and microalbuminuria, but they showed a significantly lower body weight (73.1 ± 7.6 kg vs. 76.6 ± 7.1 kg, respectively; p = 0.014), significantly lower serum levels of FPG (131.2 ± 21.95 mg/dL vs. 146.2 ± 29.5 mg/dL, respectively; p = 0.003) and a significantly lower percentage of mean HbA1c (6.5 ± 0.5% vs. 7.0 ± 0.5%, respectively; p < 0.0005) (Table 2).

**Table 2:** Intergroup analysis of metabolic and vascular variables in patients treated with dulaglutide and controls at a 3 and 9 month follow-up

Variable	Three months follow-up	Nine months follow-up
SBP (mmHg) (mean ± SD)		
Subjects treated with dulaglutide	134.9 ± 10.9	129.7 ± 10.7
Controls	133.7 ± 16.6	132.7 ± 16.7
* P*	0.653	0.264
DBP (mmHg) (mean ± SD)		
Subjects treated with dulaglutide	73.2 ± 9.9	71.8 ± 9.2
Controls	75.9 ± 9.1	75.9 ± 9.7
* P*	0.138	*0.026*
Weight (Kg) (mean ± SD)		
Subjects treated with dulaglutide	73.1 ± 7.6	68.9 ± 5.6
Controls	76.6 ± 7.1	76.3 ± 13.8
* P*	*0.014*	*0.015*
BMI (kg/m^2^) (mean ± SD)		
Subjects treated with dulaglutide	26.9 ± 3.4	25.2 ± 3.6
Controls	26.6 ± 5.0	27.8 ± 5.1
* P*	0.741	*0.003*
HR (bpm) (mean ± SD)		
Subjects treated with dulaglutide	75.6 ± 6.0	75.1 ± 5.3
Controls	78.7 ± 12.5	77.7 ± 12.5
* P*	0.097	0.161
Total cholesterol (mg/dL) (mmol/L) (mean ± SD)		
Subjects treated with dulaglutide	160.3 ± 21.2/ 4.3 ± 0.7	152.3 ± 22.4/3.94 ± 0.58
Controls	165.2 ± 26.3/4.2 ± 0.6	163.3 ± 21.5/4.22 ± 0.56
* P*	0.284	*0.009*
HDL cholesterol (mg/dL) (mmol/L) (mean ± SD)		
Subjects treated with dulaglutide	36.4 ± 4.3/0.9 ± 0.1	38.3 ± 5.7/ 0.99 ± 0.15
Controls	36.7 ± 4.3/0.9 ± 0.1	36.9 ± 4.7/0.95 ± 0.11
* P*	0.607	0.135
LDL cholesterol (mg/dl) (mmol/L) (mean ± SD)		
Subjects treated with dulaglutide	101.8 ± 10.6 /2.6 ± 0.2	96.0 ± 9.1/2.48 ± 0.24
Controls	103.8 ± 11.9/2.68 ± 0.31	103.2 ± 10.8/2.67 ± 0.28
* P*	0.339	<*0.0005*
Triglycerides (mg/dL) (mmol/L) (mean ± SD)		
Subjects treated with dulaglutide	138.1 ± 13.2/1.5 ± 0.1	137.5 ± 18.9/1.55 ± 0.21
Controls	138.0 ± 15.9/1.56 ± 0.18	138.1 ± 18.9/2.72 ± 0.42
* P*	0.971	0.878
FPG (mg/dL/mmol/L) (mean ± SD)		
Subjects treated with dulaglutide	131.2 ± 21.9/7.2 ± 1.2	119.3 ± 14.3/6.65 ± 1.05
Controls	146.2 ± 29.5/8.13 ± 1.64	145.5 ± 29.6/8.09 ± 1.64
* P*	*0.003*	< *0.0005*
HBa1C (media ± DS)		
Subjects treated with dulaglutide	6.5 ± 0.5	6.2 ± 0.3
Controls	7.0 ± 0.5	6.9 ± 0.5
* P*	<*0.0005*	< *0.0005*
GFR CKD-EPI (mean ± SD)		
Subjects treated with dulaglutide	76.7 ± 10.5	76.7 ± 9.8
Controls	76.5 ± 16.2	76.5 ± 16.2
* P*	0.954	0.959
Microalbuminuria (mg/24H) (mean ± SD)		
Subjects treated with dulaglutide	80.1 ± 16.6	79.1 ± 9.0
Controls	82.8 ± 18.5	82.5 ± 9.3
* P*	*0.432*	*0.05*
RHI (mean ± SD)		
Subjects treated with dulaglutide	1.8 ± 0.4	2.0 ± 0.4
Controls	1.8 ± 0.4	1.8 ± 0.4
* P*	0.901	*0.023*
PWV (m/s) (mean ± SD)		
Subjects treated with dulaglutide	10.8 ± 0.810.8 ± 0.8	10.6 ± 0.810.6 ± 0.8
Controls	10.9 ± 0.6	11.0 ± 0.6
* P*	0.376	*0.015*
AIx (%)( media ± DS)		
Subjects treated with dulaglutide	103.1 ± 6.7	101.8 ± 5.3
Controls	101.4 ± 3.6	101.9 ± 3.8
* P*	0.116	0.937

Data are expressed as mean (SD), n (%)

Italic emphasis indicates significant differences at statistical analysis (p < 0.05)

*SBP* systolic blood pressure, *DBP* diastolic blood pressure, *BMI* body mass index, *HR* heart rate, *LDL* low density lipoprotein, *HDL* high density lipoprotein, *FPG* fasting plasma glucose, *HBa1C* glycated hemoglobin, *PWV *pulse wave velocity, *AIX* augmentation index, *E-GFR* estimated glomerular filtration rate

P = p value of intergroup differences assessed by independent Student t test for continuous parameters

At the nine-month follow-up, the subjects treated with dulaglutide, compared with control subjects treated with traditional therapy, showed a significantly lower mean DBP value (71.8 ± 9.2 mm/Hg vs. 75.9 ± 9.7 mm/Hg, respectively; p = 0.026), body weight (68.9 ± 5.6 kg vs. 76.3 ± 13.84 mm/Hg, respectively; p = 0.015), BMI (25.2 ± 3.6 kg/m^2^ vs. 27.8 ± 5.1, respectively; p = 0.03**)**, mean total serum cholesterol (152.3 ± 22.4 vs. 163.3 ± 21.5 mg/dL 4.22 ± 0.56, respectively; p = 0.009), mean LDL serum cholesterol (96.0 ± 9.1 mg/dL vs. 103.2 ± 10.8 mg/dL, respectively; p < 0.0005), FPG (119.3 ± 14.3 mg/dL vs. 145.5 ± 29.6 mg/dL, respectively; p < 0.0005), HbA1c (6.2 ± 0.3% vs. 6.9 ± 0.5%, respectively; p < 0.0005), microalbuminuria (79.1 ± 9.0 vs. 82.5 ± 9.3 mg/dL, respectively; p = 0.05), mean PWV (10.6 ± 0.8 m/s vs. 11.0 ± 0.6, respectively; p = 0.015) and a higher mean RHI value (2.0 ± 0.4 vs. 1.8 ± 0.4, respectively; p = 0.023) (Table 2).

Intragroup analysis showed that subjects treated with once-weekly dulaglutide at the 9 month follow-up showed a significantly lower mean SBP than at baseline and the three-month follow-up, (129.7 ± 10.71 mm/Hg vs. 137.8 ± 12.1 mm/Hg vs. 134.9 ± 10.9 mm/Hg, respectively; p **<** 0.0005), mean DBP (71.9 ± 9.29 mm/Hg vs. 76.3 ± 12.2 vs. 73.2 ± 9.99, respectively; p = 0.03), weight (68.9 ± 8.8 kg vs. 73.1 ± 7.6 kg vs. 75.8 ± 8.5 kg, respectively; p **<** 0.0005), BMI (25.2 ± 3.6 kg/m^2^ vs. 26.9 ± 3.4 kg/m^2^ vs. 27.6 ± 3.4 kg/m^2,^ respectively; p **<** 0.0005), mean FPG values (119.3 ± 14.3 mg/dL vs. 147.6 ± 32.5 mg/dL vs. 131.2 ± 21.9 mg/dL, respectively; p **<** 0.0005), mean HbA1c percentage (6.2 ± 0.3% vs. 6.5 ± 0.4 vs. 7.3 ± 0.7%, respectively; p **<** 0.0005), total cholesterol (152.3 ± 22.4 mg/dL vs. 160.3 ± 21.2 mg/dL vs. 160.9 ± 20.5 mg/dL, respectively; p = 0.04), mean LDL cholesterol (96.0 ± 9.1 mg/dL vs. 102.7 ± 11.6 mg/dL vs. 101.8 ± 10.6 mg/dL, respectively; p = 0.001), mean PWV (10.6 ± 0.8 m/s vs. 11.2 ± 0.9 m/s vs. 10.8 ± 0.8 m/s, respectively; p **<** 0.0005), mean AIx (101.8 ± 5.3% vs. 105.6 ± 6.9% vs. 103.1 ± 6.7%, respectively; p = 0.001) and significantly higher median RHI values **(**2.0 ± 0.4 vs. 1.7 ± 0.44 vs. 1.8 ± 0.4, respectively; p = < 0.0005) (Table 3).

**Table 3:** Intragroup analysis of patients treated with subjects treated with conventional therapy + dulaglutide at a three and nine month follow-up

Variabile	Baseline	Three months	Nine months	p
SBP (mm/Hg) (mean ± SD)	137.8 ± 12.1	134.9 ± 10.9	129.7 ± 10.7	< 0.0005* *0.012*^
DBP (mm/Hg) (mean ± SD)	76.3 ± 12.3	73.2 ± 9.9	71.9 ± 9.2	0.03*
Weight (Kg) (mean ± SD)	75.7 ± 8.5	73.1 ± 7.6	68.9 ± 8.8	*0.02***°** < 0.0005* *0.008*^
BMI (kg/m^2^) (mean ± SD)	27.6 ± 3.4	26.9 ± 3.4	25.2 ± 3.6	< 0.0005* *0.01*^
HR (bpm) (mean ± SD)	76.6 ± 11.1	75.6 ± 6.0	75.1 ± 5.3	0.58
Total cholesterol (mg/dl/mmol/L) (mean ± SD)	160.9 ± 20.5 /4.2 ± 0.5	160.3 ± 21.2/ 4.3 ± 0.7	152.3 ± 22.4/3.9 ± 0.5	0.04*
HDL cholesterol (mg/dl/mmol/L) (mean ± SD)	37.4 ± 4.3/0.9 ± 0.1	36.4 ± 4.3/ 0.9 ± 0.1	38.3 ± 6.1/ 0.9 ± 0.1	0.79
LDL cholesterol (mg/dl/mmol/L) (mean ± SD)	102.7 ± 11.6/2.6 ± 0.3	101.8 ± 10.6 /2.6 ± 0.2	96.0 ± 9.1/2.4 ± 0.2	0.001* *0.002*^
Triglycerides (mg/dl/mmol/L) (mean ± SD)	139.2 ± 13.6/1.6 ± 0.2	138.1 ± 13.2/1.5 ± 0.1	137.5 ± 18.9/1.5 ± 0.2	0.1
FPG (mg/dL/mmol/L) (mean ± SD)	147.6 ± 32.6 /8.2 ± 1.2	131.2 ± 21.9/7.2 ± 1.2	119.3 ± 14.3/6.6 ± 1.0	*0.002*° < 0.0005* *0.001*^
HBa1C (%) (media ± DS)	7.4 ± 0.7	6.5 ± 0.5	6.2 ± 0.3	< 0.0005*°^
GFR CKD-EPI (mean ± SD)	77.4 ± 11.2	76.7 ± 10.5	76.7 ± 9.8	0.1
Microalbuminuria (mg/24 h) (mean ± SD)	80.9 ± 20.9	80.1 ± 16.6	80.1 ± 16.6	0.35
RHI (mean ± SD)	1.7 ± 0.4	1.8 ± 0.4	2.0 ± 0.4	< 0.0005*
PWV (m/s) (mean ± SD)	11.2 ± 0.9	10.8 ± 0.8	10.6 ± 0.8	*0.02*° < 0.0005*
Aix (%)(mean ± SD)	105.6 ± 6.9	103.1 ± 6.7	101.8 ± 5.3	0.001*

Data are expressed as mean (SD), n (%),

Italic emphasis indicates significant differences at statistical analysis (p < 0.05)

*SBP* systolic blood pressure, *DBP* diastolic blood pressure, *BMI* body mass index, *HR* heart rate, *LDL* low density lipoprotein, *HDL* high density lipoprotein, *FPG* fasting plasma glucose, *HBa1C* glycated hemoglobin, *PWV *pulse wave velocity, *AIX* augmentation index, *E-GFR* estimated glomerular filtration rate

P = p value of intragroup differences assessed by independent Student t test for continuous parameters

*9 months vs baseline; ^ 9 months vs 3 months; ° 3 months vs baseline

## Discussion

This study aimed to examine the efficacy in ameliorating endothelial and arterial stiffness markers of once-weekly dulaglutide (1.5 mg) plus traditional therapy in diabetic patients with type 2 diabetes.

Our study is the first to analyse the efficacy of dulaglutide therapy on surrogate vascular endpoints such as endothelial and arterial stiffness indexes in parallel with metabolic effects.

Consistent with previous studies, we demonstrate that subjects treated with traditional therapy plus dulaglutide showed significantly lower mean fasting plasma glucose and glycated haemoglobin levels at the three- and nine-month follow-ups. Thus, our study further confirms previous findings concerning the reduction of glucose serum levels and HbA1c serum levels [34, 35].

Grunberger et al. [36] demonstrated a dose-dependent HbA1c reduction for dulaglutide and greater reduction than placebo. Furthermore, the multicentre, randomized, placebo-controlled REWIND study [37] that recruited participants who aged at least 50 years with type 2 diabetes, a haemoglobin A1c (HbA1c) level of 9.5% or less, and a body mass index greater than 23 kg/m^2^ reported that the HbA1c levels were reduced in the drug group by a mean of − 0.61% compared with placebo [35].

Our study also reported that subjects treated with traditional therapy plus dulaglutide showed a significantly lower mean body weight and, BMI and significantly lower mean LDL cholesterol and total cholesterol serum levels at the nine-month follow-up.

In the REWIND study, subjects with type 2 diabetes who had a previous cardiovascular event or cardiovascular risk factors and were randomly assigned to either weekly subcutaneous injection of dulaglutide (1.5 mg) or placebo showed lower total cholesterol and lower LDL cholesterol at follow-up [37].

Atherogenic dyslipidaemia in diabetes in type 2 diabetes is characterized by the overproduction and/or delayed catabolism of triglyceride-rich particles (TRLs), including apolipoprotein apo-B-48 containing chylomicrons and apo-B100-containing VLDLs, cholesterol-rich remnant particles, small dense LDLs and a reduction in circulating HDLs [38]. When the number of postprandial TRLs is reduced in response to the induction of GLP-1 signalling, GLP-2 receptor activation is involved in the promotion of lipid absorption [39]. In experimental hamsters, an infusion of GLP1 and GLP2 causes an initial increase in lipid absorption and increased plasma concentrations of TRL-apo B48 in plasma and this response in terms of the increased postprandial lipid response has been reported to be enhanced under the condition of insulin resistance, such as in type 2 diabetes [40].

We also reported that subjects treated with traditional therapy plus dulaglutide showed significantly lower mean body weight, SBP, and DBP values at the nine month follow-up. Furthermore, at nine months, we observed a significant reduction in the levels of microalbuminuria, a well-known marker of vascular damage in diabetes [39, 40].

Regarding patients enrolled in the REWIND trial, their weight decreased by a mean of − 1.5 kg while the systolic blood pressure and LDL cholesterol levels were slightly lower in the drug group [41].

In our trial, the metabolic effects on FPG and HbA1c were observed earlier at three months. By contrast, at nine months, we observed more delayed effects on cholesterol serum variables and vascular health indexes such as RHI and arterial stiffness indexes such as PWV and AIx, possibly indicating a more strict relationship between vascular damage markers and cholesterol serum levels.

These findings are consistent with previous studies indicating the strict relationship between cardiovascular risk factors such as systolic blood pressure, total serum cholesterol and LDL levels [42–44] and cardiovascular events and vascular health surrogate markers [44].

Furthermore, as observed in our trial, and similarly in recent studies [37, 45], weekly subcutaneous injection of 1.5 mg of dulaglutide was associated with weight loss and consequently a reduction in the BMI.

A chronic inflammatory state linked to obesity leads to dysregulation of the endocrine and paracrine actions of adipocyte-derived factors, which disrupt vascular homeostasis and contribute to endothelial vasodilator dysfunction by determining, among other effects, an imbalance in the endothelin-1/nitric oxide pathway [46].

Considering these premises, the reduction of body weight and BMI observed in the dulaglutide arm at the nine-month follow-up might lead to a reduction in the systemic inflammatory state, the resumption of the correct mechanisms of vascular homeostasis and, as observed in our trial, an improvement in the vascular health indexes.

Several studies have indicated that, in addition to lowering glucose, dulaglutide affected the cardiovascular system [42–44, 47]. These investigators reported that patients with T2DM found that dulaglutide could lower systolic blood pressure by 2.8 mmHg compared with placebos. Tuttle et al., in a study of 6005 patients with T2DM, showed that dulaglutide slightly lowered the urine protein level but did not lower the glomerular filtration rate [43].

Furthermore, Ceriello et al. showed a beneficial effect of the combination of GLP-1-RA and insulin on hyperglycaemia-induced oxidative stress and endothelial function for patients with T2DM [44]. The antioxidant properties of GLP-1-RA increases intracellular antioxidant defences [47] . This mechanism may be due to the use of a GLP-1-RA affecting oxidative stress and vascular endothelial function characteristics. Another reason may be that glycaemic control is better in patients treated with dulaglutide plus conventional therapy and possibly related to an improvement in glucose variability, as reported by some studies [48].

Consistent with previous study finding [37, 49], our findings underlined the efficacy of dulaglutide on glycaemic control, as shown by its precocious action on lowering FPG and HbA1c not only at nine months but also at three months.

A previous study [48] demonstrated that the reduction of FPG and improvement in glycaemic variability are correlated with the reduction of oxidative stress on the vascular endothelium.

In particular, an increase in FPG and glucose variability can induce the worsening of oxidative stress through many mechanisms, including ROS overproduction mediated by many pathways, such as the increase in AGE levels, vasomotor imbalance mediated by the reduction of NO availability and increase in the oxidant peroxynitrite, increased inflammation molecules and overexpression of cellular adhesion molecules on the endothelial surface, such as ICAM, VCAM, and E-selectin, which promote inflammation by leukocyte rolling [50]. These pathways may contribute to endothelial dysfunction and accelerate the process of atherosclerosis, leading to increased cardiovascular events [51].

Moreover, our study showed improvement in endothelial markers such as RHI, AIx and PWV at nine months. Thus, our findings of the precocious reduction of FPG and HbA1c at three months, owing to their possible negative action on the modulation of oxidative stress, should provide an interesting explanation for the improved endothelial function, as suggested by an increase in RHI and a decrease in PWV and AIx at nine months.

Furthermore, because the treatment and placebo curves in the REWIND were separated, starting clearly between 12 and 18 months of the trial treatment period, the beneficial vascular effect of the GLP-1 receptor agonists could be due to an anti-atherogenic effect [25, 37]. Our findings are consistent with these results and may offer a pathogenetic explanation for the delayed effects on cardiovascular events, indicating the amelioration of metabolic and vascular markers observed at nine months of treatment.

Our study is the first to evaluate the beneficial effects of dulaglutide on both vascular health indexes such as arterial stiffness and endothelial dysfunction markers. These novel findings may explain the interplay between the improvement in metabolic variables and reduction in cardiovascular outcomes reported by a trial on the effect of dulaglutide [45] on cardiovascular outcomes. Our findings concerning surrogate vascular markers may also represent an explanation of the cardiovascular positive effects of other glucagon-like peptide 1 receptor agonists (GLP-1-RAs), such as liraglutide and semaglutide [49, 52–59].

Activation of the NOD-, LRR- and pyrin domain-containing protein 3 (NLRP3) inflammasome plays an important role in high glucose-induced endothelial dysfunction in patients with type 2 diabetes mellitus (T2DM). A recent study investigated whether dulaglutide possesses a protective effect against high glucose-induced activation of the NLRP3 inflammasome. These authors showed that dulaglutide treatment prevented high glucose-induced generation of reactive oxygen species (ROS) and protein carbonyls, as well as the expression of NADPH oxidase 4 (NOX-4) in human umbilical vein endothelial cells (HUVECs) [60, 61].

Another recent study compared the effect of dulaglutide and liraglutide on oxidative stress and endothelial function in patients with type 2 diabetes mellitus (T2DM). No significant differences were found in d-ROMs and logarithmic-scaled RHI (L-RHI) between the two groups after 24 weeks of treatment [62].

Glucagon-like peptide 1 receptor agonists (GLP-1 RAs) are emerging as an important therapy to consider for patients with type 2 diabetes (T2D) given the ability of this agent class to reduce glycated haemoglobin and associated weight loss and low risk for hypoglycaemia. Additionally, some cardiovascular outcomes trials (CVOTs) found non-inferiority for cardiovascular outcomes, with many findings of the superiority of these drugs.

A recent study of twelve-month treatment with GLP-1RA, SGLT-2i, and their combination showed greater improvement in vascular markers and effective cardiac cycles than insulin treatment in type 2 diabetes mellitus. The combined therapy as second line was superior to either insulin or GLP-1RA and SGLT-2i separately [63]. Another study [64] showed that 6-month treatment with liraglutide improved arterial stiffness, LV myocardial strain, LV twisting and untwisting and NT-proBNP by reducing oxidative stress in subjects with newly diagnosed T2DM.

Thus, several lines of evidence [62–66] have reported that newer antidiabetic drugs differentially affect endothelial function and arterial stiffness, as assessed by FMD and PWV, respectively. These findings could explain the distinct effects of these drugs on the cardiovascular risk of patients with type 2 diabetes mellitus.

However, most studies have evaluated the putative protective vascular role of liraglutide and only a few have evaluated this role for dulaglutide. No study has evaluated the role of both arterial stiffness and endothelial indexes; thus, our findings may appear original.

These findings have transformed our guidelines on the pharmacological treatment of T2D. Future prospective studies will be addressed to evaluate the relationship between the effects of GLP-1 RAs on vascular damage markers and the incidence of new cardiovascular events.

### Strengths and limitations

The strengths of our study include the broad and representative inclusion criteria and recruited participants, long follow-up, high retention, measurement of clinically relevant outcomes and of surrogate vascular endpoints.

A possible limitation is that we considered several endpoints (metabolic and vascular) and that we did not consider primary and secondary outcomes but some parallel aims.

## Conclusions

Our randomized trial showed that diabetic subjects treated with conventional therapy plus 1.5 mg of subcutaneous dulaglutide, compared with subjects treated with conventional therapy alone, showed favourable metabolic effects that are associated with positive effects on vascular health markers such as arterial stiffness and endothelial function markers. We furthermore reported that, unlike some metabolic effects, such as the positive effects on FPG and HbA1c, that were observed at the three-month follow-up, other metabolic effects, such as body weight and BMI reduction and positive effects on lipid serum values, were observed only at the nine-month follow-up, similarly to the positive vascular effects on PWV, AIx, and RHI.

Thus, it is intriguing to speculate that the positive vascular effects could be strictly linked to the lipidaemic metabolic positive effects of dulaglutide and reduction of body weight with consequent modulation of related adipose-derived inflammation [66].

Concerning this issue, further studies will be addressed by our group to evaluate the possible effects of dulaglutide treatment on adipose-derived hormones and cytokines, such as PAI-1, adiponectin, and IL-6, that may be favourably changed by dulaglutide treatment. Nevertheless, long-term dulaglutide use might reduce clinically relevant ischaemic stroke in people with type 2 diabetes [67]. This result, since to the not direct relationship between serum lipid levels and stroke risk may suggest possible direct vascular effects of dulaglutide, maybe more relevant on the setting of the extracranial and intracranial brain circulation.

## Data Availability

All data and material are available on figshare (https://figshare.com/s/2fa3227c64b263f9b7cf).

## Abbreviations

CV: Cardiovascular
GLP: Glucagon-like peptide 1
FPG: Fasting plasma glucose
HbA1c: Glycated haemoglobin
LDL: Low-density lipoprotein
HDL: High-density lipoprotein
BMI: Body mass index
FMD: Flow-mediated vasodilation
RH-PAT: Reactive hyperaemia peripheral arterial tonometry
NAFLD: Non-alcoholic fatty liver disease
eGFR: Estimated glomerular filtration rate
ESC-ESH: European Society of Cardiology-European Society of Hypertension
BP: Blood pressure
AIx: Augmentation index
PP: Pulsatory pressure
RHI: Reactive hyperaemia index
PWV: Pulse wave velocity
NLRP3: NOD-, LRR- and pyrin domain-containing protein 3
SBP: Systolic blood pressure
DBP: Diastolic blood pressure
TRLs: Triglycerides-rich particles
VLDL: Very-low-density lipoprotein
T2DM: Type 2 diabetes mellitus
GLP-1-RA: Glucagon-like peptide 1 receptor agonist
ROS: Reactive oxygen species
AGEs: Advanced glycation end-products
NO: Nitric oxide
ICAM-1: Intercellular adhesion molecule 1
VCAM-1: Vascular cell adhesion molecule 1
CVOTs: Cardiovascular outcomes trials
